# Early Versus Late Weight-Bearing After Ankle Fracture Surgery: A Comparative Review

**DOI:** 10.7759/cureus.97417

**Published:** 2025-11-21

**Authors:** Bishr Elsharydah, Sarah N Powell, Urvaksh Avanthsa, Zachary Shorts, Hannah Sudhakar, Megan J Blount, Ivan Marrufo, Irina D Sokolik

**Affiliations:** 1 College of Osteopathic Medicine, Kansas City University, Kansas City, USA; 2 Orthopedics, University of Nebraska Medical Center, Omaha, USA; 3 Medicine, Kansas College of Osteopathic Medicine, Wichita, USA; 4 Orthopedics, A.T. Still University School of Osteopathic Medicine, Mesa, USA; 5 Medicine, University of New England College of Osteopathic Medicine, Biddeford, USA; 6 Orthopedics, University of California Davis Health System, Sacramento, USA; 7 Public Health, University of North Texas Health Sciences Center-Texas College of Osteopathic Medicine, Fort Worth, USA; 8 College of Medicine, State University of New York Upstate Medical University, Syracuse, USA

**Keywords:** ankle fracture, complications, early weight bearing, functional outcomes, late weight bearing, open reduction and internal fixation, orthopedic surgery

## Abstract

Early weight-bearing (EWB) and late weight-bearing (LWB) protocols are the two primary regimens implemented for patients following ankle fracture surgery, yet their outcomes remain variable. This review aimed to assess these protocols, including their limitations and implications, to support more tailored postoperative management and ultimately improve patient recovery. EWB demonstrated superior short-term outcomes compared to LWB, including higher functional assessment scores, improved range of motion, and earlier return to work times. However, patients in this group revealed lower protocol adherence and higher wound-related issues in select patients, including complication and reoperation rates. As a result, LWB is more often favored by surgeons for elderly, diabetic, or morbidly obese patients despite lower early functional outcome scores. Overall, the literature remains inconclusive, highlighting the need for personalized rehabilitation plans and further research to refine risk stratification and optimize recovery across diverse patient populations.

## Introduction and background

Ankle fractures are among the most common orthopedic injuries, currently ranking as the third most common type of fracture in orthopedic practice [1,2]. Depending on fracture morphology, surgical intervention may be required. Following open reduction and internal fixation (ORIF), postoperative weight-bearing protocols are implemented. These protocols can range from early weight-bearing (EWB) or late weight-bearing (LWB) protocols to optimize fracture healing while avoiding fracture displacement or implant failure, as well as help mitigate postoperative complications such as stiffness and wound breakdown [1,2].

Despite the widespread use of different postoperative weight-bearing restrictions, opinions remain inconsistent regarding their effectiveness in delivering optimal postoperative care [3]. This review outlines and compares protocols following ankle fracture surgery, including clinical recovery scores, functional outcomes, complication rates, and other patient-related variables. Among many other equally important considerations, selecting the most appropriate protocol requires a holistic approach to optimize patient recovery and positively impact patient outcomes.

Surgical indications and techniques

Surgical intervention is primarily indicated for fractures that are unstable, displaced, and/or irreducible by closed means. Radiographic signs include talar shift or medial clear space widening. Unstable isolated lateral malleolar fractures with deltoid ligament or syndesmotic rupture also require surgery [4]. Additional indications include a variety of failures of a closed reduction, open fractures, syndesmotic injuries demonstrating instability, or injuries with soft tissue interruption. Open fractures or those with neurovascular compromise require urgent operative management [5]. These criteria help guide surgical decision-making to optimize the alignment, joint stability, and long-term functional outcomes. 

The specific approach for fixation is determined by the fracture morphology [6]. ORIF typically includes fixation involving plates and screws, with the addition of syndesmotic fixation when there is syndesmotic instability [7]. In some cases, variations of fixation may be used. For example, an intramedullary fixation of the fibula (fibular nails) can be used in place of a lateral plate for distal fibula fractures [8]. In some severe fractures and dislocations or cases that may have compromised soft tissue (open fractures or extensive swelling), external fixation or staged surgery may be necessary before ORIF is definitively performed following soft tissue convalescence [9]. Overall, different fracture patterns influence the approach for fixation, and these variations in methods can influence the postoperative protocols. Ultimately, the specific surgical method chosen may completely vary based on the fracture type and patient factors, with the goal in each situation to restore the ankle joint mortise and stable fixation.

Postoperative weight-bearing protocols

Definitions of Weight-Bearing Protocols

Postoperative weight-bearing protocols following ankle fracture surgery are generally described as either EWB or LWB. For the purpose of this review, two categories will be used: EWB will refer to weight-bearing initiated before six weeks postoperatively, while LWB will refer to weight-bearing initiated after six weeks. All differences in immobilization practices will be addressed within their respective sections.

Outcome Measures

Comparative studies commonly use validated outcome measures to assess recovery, such as the American Orthopaedic Foot & Ankle Society (AOFAS) Ankle-Hindfoot Score, the Olerud Molander Ankle Score (OMAS), time to return to work, and time to return to pre-injury activities. The AOFAS Ankle-Hindfoot Score assesses pain, alignment, and functionality through a score of 0-100, with a higher number reflecting better outcomes [10]. OMAS is a patient-reported score of 0-100 measuring pain, stiffness, swelling, stair climbing, running, jumping, squatting, and the use of supports, with a higher score here showing better function. In addition, many studies employ the SF-36 or SF-12, a validated general health questionnaire that reports physical and mental competency scores (normalized to a population mean of 50, SD 10; higher scores indicate better health status) across eight domains, capturing both physical and mental aspects of recovery. Another scale that is commonly used is the Barthel Index score, which is a scale that correlates with a patient's ability to perform basic activities of daily living. However, it is important to consider that each of these measures has its own validative limitations, which should be considered when interpreting results across studies.

Further Considerations

In addition to the timing of weight-bearing, postoperative protocols including the usage of removable ankle supports, such as walking boots or braces, may offer early improvement in function and quality of life when compared to immobilization [11]. However, they might also be associated with a higher risk of wound complications when compared to immobilization with splints or casts [12]. Wound healing generally marks the point at which early range-of-motion exercises can be safely introduced; beginning such exercises too soon may increase complication rates without offering clear functional benefits [13]. These different considerations show just how important individualized and subjective rehabilitation strategies may be in balancing the benefits and potential drawbacks between early and late weight-bearing.

## Review

Early weight-bearing protocols

Clinical Outcomes

EWB protocols, defined as weight bearing within six weeks postoperatively, present multiple potential advantages. The Weight-Bearing in Ankle Fracture (WAX) trial, a multicenter randomized control trial of 561 patients, found higher OMAS scores at six weeks in the EWB group (47.3 vs. 38.7; adjusted mean difference 8.51; 95% CI: 4.72-12.30; p < 0.0001), but no significant difference at one year, with early differences falling below the minimal clinically important differences (76.5 vs. 72.4; adjusted mean difference 3.72; 95% CI: -0.18-7.64; p < 0.062) [14]. Similarly, a meta-analysis of several RCTs examined outcomes following EWB, such as return to work, quality of life (assessed by the SF-36), complications, and OMAS [15]. One of the RCTs defined EWB as ambulating in a walking cast immediately after surgery, while the other RCTs stabilized the ankle with a plaster splint for 2-14 days before moving to a weight-bearing cast, with LWB being defined at six to seven weeks postoperatively [15]. There was a significant difference (95% CI: 0.46-5.86, p = 0.02) in OMAS at 12 weeks with improved early function in the EWB group compared to the LWB group, but no difference at one year [15]. There was not a significant difference in days to return to work in four of the studies, but two of them did state that the EWB group took fewer days to return to work (53.8 in EWB vs. 106.5 in LWB, p = 0.007) [15]. SF-36 scores from the included studies also favored EWB.

Regarding complications across the studies, 86 of 600 patients experienced a complication, primarily wound-related, with 54 in the EWB group and 19 in the LWB group, and the remainder from unspecified treatment groups [15]. The EWB group did have more cases of wound-related complications than the LWB group, but of importance, one study accounted for 56% of all reported complications, and if this study was removed, there would be no statistically significant difference in wound complications between the groups [15]. This large study indicates that there are advantages to EWB when it comes to early OMAS measurements, days to return to work, and quality of life, but an increase in complications in the EWB related to wound healing. Another RCT assessed OMAS at 12 months postoperatively, which also found that EWB was noninferior to LWB, and the time taken to return to physical activity was less in the EWB than LWB (9.1 ± 3.0 vs. 11.0 ± 3.0 weeks; p < 0.001) [16]. Together, these two studies suggest that patient-reported outcomes are initially higher for EWB and that EWB can be clinically non-inferior to LWB. These findings help support the consideration for EWB in most surgically treated ankle fractures, considering that the fixation is secure and there are no complicating factors, such as traumatic soft tissue injury or poor bone quality. 

Objective Functional Outcomes

Beyond patient-reported outcomes, the differences with regard to objective outcomes have also been studied between EWB and LWB protocols. An early prospective study showed there was no significant difference between LWB and EWB groups in regards to swelling, muscle wasting, or pain [17]. Similarly, in another early prospective study of 43 patients comparing EWB versus LWB, there was no significant difference in pain ratings between the two groups [18]. Furthermore, in an RCT that compared EWB to LWB groups after surgery, there was greater improvement in the flexion/extension arc of motion in the EWB than the LWB at six weeks (41° in EWB vs. 29° in LWB, p < 0.0001) [19]. In a systematic review of nine studies (n = 555), there were significantly improved outcomes for early dorsiflexion (within three months) [17]. Lastly, in a more recent systematic review of functional outcomes and safety of EWB, there was significantly greater pain reduction in the EWB group compared to the LWB group (standardized mean difference +0.32, 95% CI: 0.21-0.43) and ankle dorsiflexion (standardized mean difference: +0.38, 95% CI: 0.26-0.50) [20]. The EWB in this study also had lower complication risk (RR: 0.89, 95% CI: 0.69-1.14) and fewer immobilization-related complications compared to LWB, such as deep vein thrombosis (DVT): 2.5% vs. 6.3% and CRPS 1.8% vs. 4.7% [20].

Overall, these studies demonstrate that there can be multiple potential upsides to EWB, including greater early ankle range of motion, quicker return to work, and earlier improvements in patient-reported outcome measures (PROMs). This must be measured against the possibility of an increased risk of wound healing complications demonstrated with EWB protocols. Similarly, some authors have suggested an association with a higher risk of wound complications when compared to immobilization with splints or casts [12]. Importantly, wound healing is thought to be the general starting point of when early range of motion exercise can be implemented, where the initiation prior may lead to an increase in complications without a clear functional benefit [13].

Complications and Other Considerations

Several limitations regarding EWB remain regarding its broader application. In a comparison between LWB with a short leg cast and EWB with functional ankle brace mobilization protocols, Lehtonen et al. found that EWB had a significantly higher percentage of wound complications (66% vs. 16%, p = 0.0005) [21]. Additionally, while the early range of motion before wound healing does not significantly increase infection rates, it is associated with a 3.11-fold increase in the odds of complications, with superficial wound healing being the most common. (95% CI: 1.64-5.90) [13]. Furthermore, despite the absence of direct comparative studies between EWB and LWB following ORIF in patients with syndesmotic injuries, Lazarow et al. observed reoperation rates ranging from 9% to 31% in three studies of EWB, vs. 0% to 11% in studies of LWB [22]. 

While EWB improves patient independence, it is associated with decreased compliance. Braun et al. found that only 47% of patients adhered to recommended weight-bearing guidelines, with deviations from the recommendation exceeding 50% within two weeks after ORIF via real-time pedobarography monitoring. Patients in the EWB demonstrated a significantly greater deviation from recommendations (p = 0.01) [23]. On the other end of the spectrum, concerns about falling as well as perceived frailty are common among older populations, impeding patient confidence in applying weight and diminishing the benefits of utilizing EWB after ORIF [24]. Negative setbacks such as pain, swelling, or low morale may impede patient confidence in recovery utilizing EWB protocols [25]. These findings highlight that EWB success depends on careful patient selection, fracture stability, and addressing psychological and physical barriers to adherence.

Late weight-bearing protocols

Objective Functional Outcomes

Contrasting the mobility-focused approach of EWB protocols, LWB protocols aim to decrease the theoretical risks of fixation failure and wound complications [26]. Khojaly et al. performed a randomized controlled trial comparing EWB in a walking boot to LWB. As with other trials, the EWB group demonstrated a higher mean OMAS score at six weeks (difference in means 10.4; p = 0.005) and three months (difference in means 12.0; p = 0.003) compared to the LWB group, although these differences diminish at six months and one year [6]. Therefore, the EWB approach seems to confer an earlier return-to-function for patients, although effects between the groups diminish later in the healing process. Additionally, there was no significant difference in the rate of postoperative complications between the EWB and LWB groups in this study (OR: 1.09; p = 0.863) [6]. Analyses of RAND-36, a validated quality of life questionnaire similar to the SF-36, scores between the EWB and LWB group showed no significant differences in score for physical health or mental health at two weeks, six weeks, three months, six months, and one year postoperatively [6]. However, at six weeks, 82% of the EWB patients had returned to work, compared to 39% of the other groups (OR: 7.05; p < 0.01), indicating potential increased financial burdens for patients with an LWB protocol [6].

Limitations and Comparative Studies

Similar studies echo the larger conclusion that, in terms of functional outcomes, utilizing a six-week LWB protocol does not seem to offer superior results and could be slowing recovery [27]. Smeeing et al. found that, in a study of 115 adult patients, the unprotected weight-bearing group had a significantly higher ankle function score at six weeks compared to other weight-bearing groups, including protected weight-bearing and non-weight-bearing. Furthermore, the unprotected weight-bearing group had a significantly earlier return to work and sports with no difference in complication rates [27]. Chen et al. found that patients with LWB recovered functional mobility significantly slower in the short term compared to EWB at six weeks (SMD = 0.69, 95% CI: 0.49-0.88 and p < 0.01), 12 weeks (SMD = 0.57, 95% CI: 0.22-0.92 and p < 0.01), and 24-26 weeks (SMD = 0.52, 95% CI: 0.20-0.85 and p < 0.01) post-surgery [28]. Additionally, LWB was found not to significantly reduce complications such as infections or wound issues compared to EWB, while also possibly increasing the risk of disuse syndrome, or stiffness due to prolonged immobilization [28]. Due to there being conflicting data regarding complications and the potential for poorer short-term outcomes in PROMs, LWB may provide little benefit over EWB for operative ankle fractures [29]. Compared to EWB, LWB showed significantly lower functional improvements at the six-week and 12-month periods post-ankle fracture surgery [30]. 

Practice Variation and Surgeon Considerations

Variation and lack of standardization in postoperative protocols can not only create confusion for patients but can also make it more difficult to objectively study outcomes [26]. While injury pattern and medical status were the most frequently cited factors, standardized guidelines for implementing LWB after ankle fractures remain ambiguous [26]. These findings highlight a need for clear, evidence-based protocols to guide weight-bearing progression after ankle fracture surgery, in addition to more randomized controlled trials comparing the efficacy of LWB to other weight-bearing strategies.

Surgeons often consider several patient-specific factors when prescribing a treatment/weight-bearing protocol for patients after ankle fracture surgery. A cross-sectional expert opinion survey of AOFAS and Orthopaedic Trauma Association (OTA) members to determine how long they instruct patients to be non-weight-bearing (NWB) following ankle ORIF [26]. 702 surgeons were interviewed to determine the difference in prescribed late or non-weight-bearing periods between younger and older healthy patients, as well as older patients with comorbidities [26]. Consistently, across different types of fractures, surgeons prescribed longer periods (in weeks) of NWB to older healthy patients than younger patients with the same type of ankle fracture, and older patients with medical comorbidities received the longest periods of NWB [26]. Surgeons were also asked what factors played a role in determining how long a patient should be NWB, and cited factors such as bone quality (79%), medical comorbidities (77%), type of fracture (64%), presence of osteoporosis (50%), and age of patient (43%) [26]. Thus, poor bone quality and medical comorbidities impact weight-bearing recommendations following ankle fracture surgery. These factors could pose a barrier to surgeons ' willingness to implement EWB protocols in their practice.

Special considerations for weight-bearing protocols

Diabetic Patients

One particular medical comorbidity that has been researched with regard to ankle fractures and weight-bearing status is in patients with diabetes [31]. This patient population is at a high risk for wound healing complications secondary to microvascular disease, and their postoperative course can be complicated by neuropathy if present; therefore, it may be of benefit in this population to protect weight-bearing for longer periods of time. While this retrospective cohort study found that there were fewer wound complications associated with LWB (>8 weeks in this study), the data were not statistically significant when compared with EWB (29.2% vs. 39.4%, p = 0.37) [31]. Additionally, while this study did demonstrate higher rates of hardware failure (EWB vs. LWB, n = 8 vs. n = 1), revision (EWB vs. LWB, n = 14 vs. n = 3), revision for hardware failure (EWB vs. LWB, n = 5 vs. n = 0), and nonunion (EWB vs. LWB, n = 7 vs. n = 1), these were not statistically significant differences in their cohort [31].

Conversely, Bazarov et al. examined the efficacy, risk factors, and complications of EWB in patients with diabetes after operative and non-operative closed ankle fractures [32]. In this study, all patients were able to progress with protected weight bearing in a cast or removable walking boot at two weeks after surgery. The authors found that wound dehiscence was the most common complication in operative ankle fractures (18.8%, 9/48 patients), and complications were far more common in patients over the age of 60 (91.7% vs. 8.3%, p = 0.0403) [32]. This may be due to older patients being at increased risk for further disease progression and other comorbidities that impact healing after injury and surgery. Both studies by Brutico et al. and Bazarov et al. are limited by their sample sizes, making it difficult to generalize their results to the greater population.

In patients with diabetes, it is important to stratify disease severity and how that impacts the healing process. Wukich et al. investigated how outcomes compared between patients with and without end-organ damage secondary to diabetes [33]. It was ultimately determined that individuals with complicated diabetes had a 3.8-fold increased risk of overall complications (p = 0.003), with non-infectious complications being 3.4 times more likely when compared to individuals with uncomplicated diabetes (p = 0.02) [33]. The authors stressed the importance of preoperative assessment of end-organ damage in patients with diabetes, like peripheral neuropathy or vascular disease, as there is the potential for these patients to not be aware of the extent of their disease [33]. 

Elderly and Obese Patients

Elderly and obese patients present unique challenges regarding postoperative regimens, likely due to concerns surrounding poor bone quality and increased mechanical load stress, respectively. Despite these concerns, even recent literature investigating the effects of LWB duration in geriatric ankle fractures is sparse [34]. However, several studies and systematic reviews have found that EWB may impart both short and long-term benefits without increasing the rate of complications in older adults post-ankle fracture surgery [34,35,36]. For example, Lorente et al. compared 70 elderly patients treated with early and late or NWB groups. Patients in the early weight-bearing group had significantly higher SF-12 scores, in both the short and long term (64.9 vs. 52.9; p < 0.001 and 81.0 vs. 69.8; p < 0.001), as well as higher Barthel Index scores (64.2 vs. 54.3) [35]. Most importantly, there were no significant differences in complications between the groups [35]. In addition, Van Halsema et al. concluded that, although evidence is limited in elderly patients, permissive early weight bearing appears safe in relatively healthy elderly patients without increasing complications [36].

Moreover, restrictive weight bearing in patients over 70 years old has been shown to reduce their ability to maintain independent living, which could significantly impact quality of life [8]. Thus, when considering treatment protocol for older patients undergoing ankle fracture surgery, the literature suggests that encouraging weight bearing and mobility as early as possible does not confer an increased risk of complication, but may offer an improved quality of life, return to function, and earlier resumption of independent activities of daily living. Furthermore, in a retrospective study that included patients only over the age of 65, EWB groups were associated with a lower discharge rate to rehabilitation facilities (25% vs. 90%, p = 0.0036) without an increase in wound complications [37]. This suggests that, even in elderly patients, EWB may allow more patients to be discharged directly home, without fear of any wound healing complications.

Morbidly obese patients are another population often approached with caution when considering weight-bearing protocols, although there is a lack of literature investigating the optimal duration of immobilization for obese patients following ankle fracture surgery. Obese patients are at an increased risk of more significant ankle fractures involving the syndesmosis, as well as displaced fractures, and are thus more difficult to treat, increasing surgeon hesitancy for early mobilization [38]. However, obese patients are also at a heightened risk for immobilization-related complications, such as DVT and pulmonary embolism (PE), local skin infections due to moisture within the cast and fluctuant edema, and pressure-related injuries [38]. Removable leg braces allow for mobilization exercises and ease of skin inspection, but also likely contribute to patient non-compliance with LWB protocols, increasing the risk of loss of reduction in the early stages of healing [38]. Thus, a combined approach may be best for patients with obesity, combining a removable cast to facilitate mobilization exercises and maintain skin health while also educating patients to adhere to a strict LWB period to facilitate healing of the ankle joint. 

Socioeconomic and Cost Implications

Few studies have quantified and compared the direct and indirect costs between EWB and LWB. Additionally, there is limited data on the economic burden difference between different populations. Management of ankle fractures can impose a significant financial burden on patients, stemming from prolonged recovery time, potential reoperation, and the need for extended rehabilitation. The WAX trial [14], a multicenter randomized controlled trial, investigated the financial benefit of EWB versus LWB protocols post-surgical fixation over 12 months. The study found that EWB had a greater cost-benefit and improved Quality-Adjusted Life Years (QALYs) compared to LWB. These benefits extended beyond direct healthcare costs, including indirect socioeconomic impacts such as shorter rehabilitation duration and quicker return to work. Notably, EWB was not only more economically favorable in this study, but was also associated with favorable functional scores with no significant changes in complication rates. These findings may indicate additional reasons to consider EWB over LWB in appropriately selected patients [14,39,40,16].

## Conclusions

With ankle fractures being one of the most common types of fractures worldwide, postoperative weight-bearing protocols are crucial for supporting optimal patient recovery. Although each approach carries its own advantages and drawbacks, there is still no universally accepted protocol for postoperative management after ankle fracture surgery. Given their widespread uses in postsurgical settings, each weight-bearing protocol is examined thoroughly in order to evaluate those differences. Current literature suggests that EWB after ankle fracture surgery may offer promising advantages for select patients. EWB has been associated with early improvements in OMAS and Ankle-Hindfoot Scores, better early functional outcomes, faster return to physical activity, and greater gains in range of motion compared with LWB. However, EWB has also been linked to lower protocol adherence, higher rates of wound complications, and increased reoperation rates.

Additional studies indicate that surgeons often choose LWB for elderly patients because of advanced age and reduced bone density. Similar trends are seen in morbidly obese and diabetic patients, whose medical complexity and heightened risk of wound complications make LWB a more conservative choice. However, LWB groups demonstrated overall lower OMAS scores in comparison to EWB groups at earlier postoperative times, but was insignificant by the one-year mark. In some studies, LWB groups also were shown to have delays in return to work and return to physical activity timeframes. Noting this, it is critical for surgeons to weigh these factors into consideration; while LWB may seem more appropriate for older or higher-risk populations, they could also pose additional risks, and some evidence suggests EWB protocols may still be safe and beneficial in select elderly patients. Despite the advantages observed with EWB, the current literature remains inconclusive, as patient-specific factors continue to play a central role in determining optimal outcomes. This underscores the need for further research aimed at refining patient risk stratification to guide postoperative rehabilitation protocols that best support fracture healing and recovery.
